# Case Report: Complex venlafaxine overdose and prolonged intensive care unit hospitalization associated to combined CYP2D6 and CYP2C19 polymorphisms

**DOI:** 10.3389/fpsyt.2026.1943247

**Published:** 2026-08-26

**Authors:** Bruno Michel, Lidvine Boland, Ludovic Gérard, Virginie Montiel, Vincent Haufroid, Joao Pinto Pereira

**Affiliations:** 1 Department of Clinical Pharmacy, Cliniques universitaires Saint-Luc, Brussels, Belgium; 2 Department of Clinical Chemistry, Cliniques universitaires Saint-Luc, Brussels, Belgium & Louvain centre for Toxicology and Applied Pharmacology (LTAP), Institut de Recherche Expérimentale et Clinique, UClouvain, Brussels, Belgium; 3 Department of Intensive Care, Cliniques universitaires Saint-Luc, Brussels, Belgium

**Keywords:** cardiogenic shock, CYP2C19, CYP2D6, hypoglycaemia, pharmacobezoar, pharmacogenomics, serotonin syndrome, venlafaxine

## Abstract

**Introduction:**

Venlafaxine (VEN) undergoes extensive hepatic metabolism, mainly via CYP2D6. Other minor pathways are also involved in VEN metabolism, some of which have clinical significance, such as the CYP2C19 pathway. Genetic variations in these can impact metabolism, increasing toxicity risk or reducing the medication’s efficacy. While VEN is generally well tolerated, overdose can lead to severe complications, including cardiotoxicity, hypoglycaemia, serotonin syndrome, and seizures, often requiring intensive care unit (ICU) management.

**Case report:**

A 73-year-old female patient was admitted to the emergency department in an unconscious state following a suspected intentional overdose of VEN. Shortly after her admission the patient was transferred to the intensive care unit due to multiorgan failure where she remained for 30 days. Serial VEN level measurements were conducted along with pharmacogenetic testing that revealed a CYP2C19 *poor metabolizer* and a CYP2D6 *intermediate metabolizer* profile, which contributed to high VEN plasma levels that influenced the patient’s prolonged recovery and clinical course.

**Conclusion:**

Genetic polymorphisms affecting VEN metabolism contributed to higher plasma concentrations exceeding 20,000 ng/mL and, subsequently, to a higher risk of toxicity. The patient developed serotonin syndrome, hypoglycaemia and cardiotoxicity, resulting in cardiogenic shock. A rare combined *CYP2D6/CYP2C19* metabolism impairment may have contributed to the severity while age and potential pharmacobezoar formation may further have worsened toxicity and clinical course. This case underscores the importance of early plasma monitoring and pharmacogenetic assessment in overdose management, as a rare metabolic profile affecting VEN clearance contributed to prolonged toxicity and complex ICU management. To our knowledge, this is one of the first cases to describe the potential clinical impact of a rare combined CYP2D6/CYP2C19 polymorphism.

## Introduction

Venlafaxine (VEN) is a serotonin–norepinephrine reuptake inhibitor with mild dopaminergic activity that undergoes extensive hepatic metabolism. CYP2D6 is the primary enzyme involved, generating the major active metabolite O-desmethylvenlafaxine (ODV; Desvenlafaxine), while CYP2C19 plays a secondary but clinically relevant role. N-desmethylvenlafaxine (NDV) is a less active metabolite formed mainly via CYP3A4 and CYP2C19, which also contribute to ODV formation to a lesser extent. Pharmacokinetic modeling and therapeutic drug monitoring studies show that CYP2D6 poor metabolizers have reduced VEN clearance, resulting in increased drug exposure and higher VEN/ODV metabolic ratios, with CYP2C19 intermediate metabolizers showing similar but less pronounced effects. Together, CYP2D6 and CYP2C19 phenotypes account for approximately 46% of interindividual variability in dose-adjusted VEN concentrations, compared with 24% for CYP2D6 alone (1–3). Genetic variation in one or more cytochrome enzymes involved in VEN and ODV metabolism may predispose patients to increased VEN exposure and toxicity (4).

The clinical impact of combined CYP2D6 and CYP2C19 impaired metabolism in VEN overdose remains poorly documented.

The main aim of this report is to describe the complicated and prolonged intensive care management of a rare case of massive VEN extended-release (XR) overdose in a patient exhibiting a combined CYP2D6 *intermediate metabolizer* and CYP2C19 *poor metabolizer* phenotype.

This contributed to dangerously high and prolonged VEN exposure, resulting in multiorgan failure and an extended stay in the intensive care unit.

This case report was written in accordance with the CARE guidelines (5).

## Case

A 73-year-old female patient was admitted to the emergency department in an unconscious state following a suspected intentional VEN overdose.

Upon initial assessment by first responders, the patient exhibited a Glasgow Coma Scale (GCS) score of 10/15, hypotension, and generalized hypertonic state with clonic movements affecting all four limbs. She required supplemental oxygen at 2 L/min to maintain a transcutaneous oxygen saturation of 95%. Electrocardiography revealed a prolonged QTc interval (553ms) with an intermittent right bundle branch block (240ms).

The patient’s medical history included ischemic heart disease, and a known diagnosis of anxiety disorder, with two previous suicide attempts. Her known chronic medication was composed of acetylsalicylic acid 80 mg, simvastatin 20 mg and VEN XR 150 mg. At her residence, 60 empty blisters of VEN XR 150 mg (suggesting an estimated ingestion of approximately 9 g) and 10 empty blisters of 2 mg bromazepam (i.e. 20 mg) were discovered.

Eight hours post-ingestion, following hospital admission, the patient’s condition rapidly deteriorated, with hypotension unresponsive to fluid resuscitation, bradycardia (<60bpm), and a decline in consciousness (GCS of 6/15), requiring intubation and vasopressor support (norepinephrine) to manage hemodynamic instability. Due to the 8-hour delay post-ingestion, no activated charcoal or gastrointestinal decontamination was performed, and whole-bowel lavage was avoided given hemodynamic instability.

A transthoracic echocardiogram revealed a diffuse biventricular dysfunction (with a reduced left ejection fraction of 30% calculated by Simpson biplane method and a TAPSE of 0.90 cm), necessitating the initiation of inotropic support using milrinone and dobutamine. By 14 hours post-ingestion, the patient developed acute kidney injury with marked oliguria (urine output <250 mL in 24 hours). Her respiratory function remained relatively stable, requiring a fraction of inspired oxygen (FiO2) of 35%.

By days 2-3, hemodynamic parameters gradually improved, allowing tapering of dobutamine and milrinone, and her renal function began to recover with increased urine output. VEN was detected in gastric fluid aspirated on day 3.

The patient’s level of consciousness improved allowing extubation on day 3.

Unfortunately, six hours after extubation, the patient required re-intubation on day 4 due to refractory hypoxemia, secondary to ventilator-associated pneumonia and cardiogenic oedema linked to a paroxysmal episode of atrial fibrillation with high ventricular response. In response, the patient was placed on diuretic therapy and placed in prone position to optimize oxygenation.

The clinical course was further complicated by episodes of recurrent hypoglycaemia (resistant to repeated administrations of 6 grams of intravenous glucose over the course of 3 days) which resolved after the initiation of enteral feeding through a nasogastric tube and trials of octreotide administration, consistent with rare reports in the literature (6). Repeated sedation-weaning trials failed to achieve meaningful interaction with the patient. Extensive neurological evaluations, including EEG, brain CT, and MRI, were unable to identify any other underlying cause for her persistent altered mental state.

**Figure 1:**
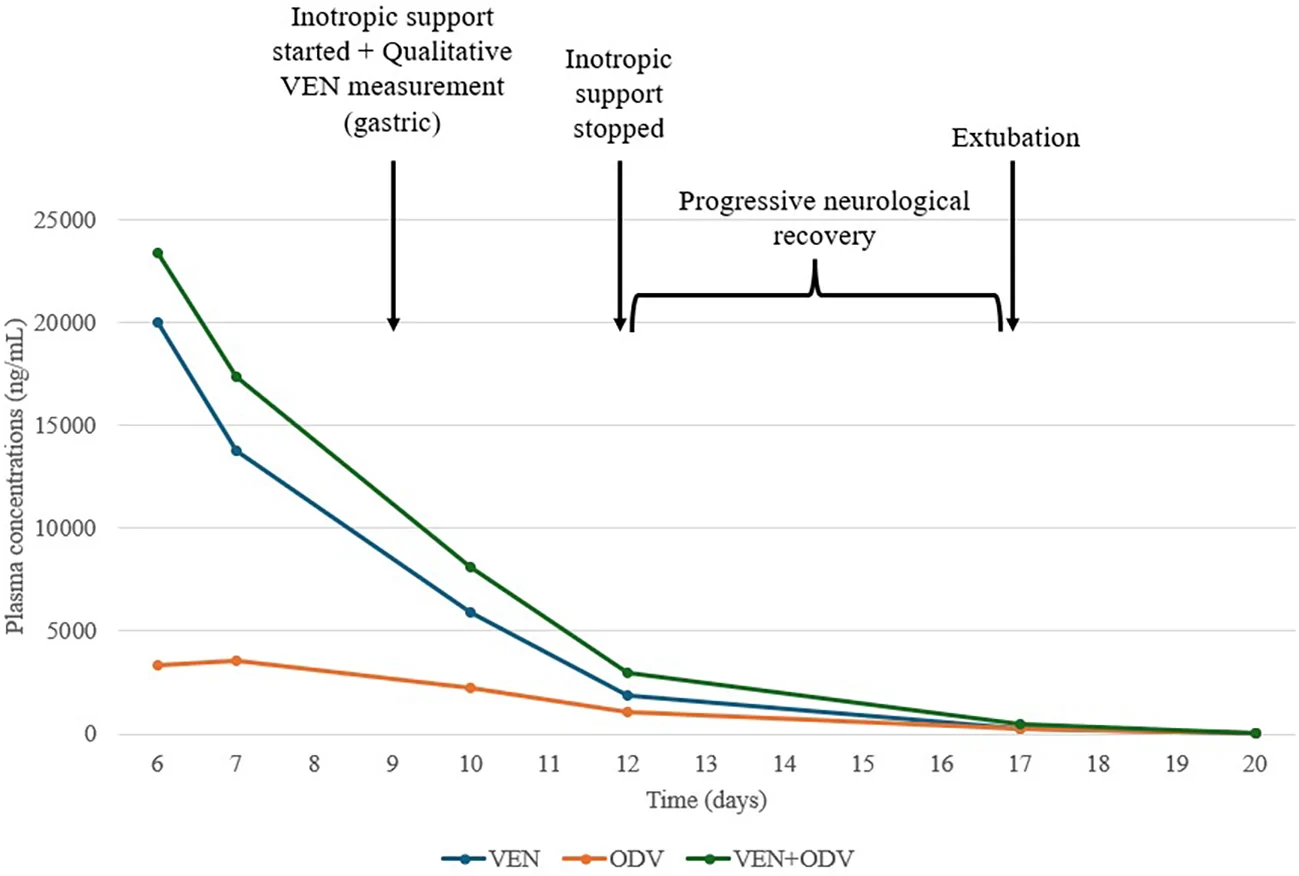
Evolution of venlafaxine (VEN) and O-desmethylvenlafaxine (ODV) plasma concentrations over time with potential clinical correlations. The first blood sample for quantitative analysis was collected 147 hours after ingestion (LOQ_VEN_ = 4 ng/mL and LOQ_ODV_ = 6 ng/mL).

Serial blood samples were collected starting on day 6 for the measurement of VEN and ODV concentrations. Quantification was performed using liquid chromatography coupled with tandem mass spectrometry (LC-MS/MS). The highest plasma levels observed were 20,031 ng/mL for VEN and 3,341 ng/mL for ODV, whereas the therapeutic reference range for combined VEN + ODV concentrations is 100-400 ng/mL. All measured concentrations and potential clinical correlations are presented in **Figure 1**. Bromazepam was not quantified.

On day 9, a single inotropic support by dobutamine was restarted due to recurrent biventricular failure, although it was progressively completely weaned off 72 hours later. VEN was again detected in gastric fluid aspirated on day 9.

Afterwards the patient began to demonstrate progressive neurological recovery, with intermittent responses to simple commands and sustained eye contact, allowing for another successful extubation on day 17. Despite these improvements, the patient remained disoriented for the following 20 days.

Given the patient’s slow clinical course and VEN and ODV concentrations markedly elevated even on day 6-far beyond what would normally be expected after an acute overdose-, a polymorphism affecting VEN metabolic pathways was suspected. Pharmacogenomic testing was performed on day 20 using SOPHiA DDM^®^ for Pharmacogenomics on a MiSeq Illumina Inc. (United States). This analysis revealed, on one hand, a CYP2D6 *intermediate metabolizer* (CYP2D6*5/*27) status, characterized by a complete loss of an allele activity (CYP2D6*5) and a normal activity of another allele (CYP2D6*27), and, on the other hand, a CYP2C19 *poor metabolizer* (CYP2C19*2/*2 – complete loss of both alleles’ activity) status.

Regarding the other CYP enzymes that play a less significant role in VEN metabolism, the analysis showed a normal CYP3A4 metabolizer status (CYP3A4*1/*1) and an intermediate CYP2C9 metabolizer status [(CYP2C9*1/*12, with heterozygous presence of c.1465C>T variant, rs9332239)].

## Discussion

We report the case of a 73-year-old woman admitted to the ICU for intentional VEN and bromazepam overdose, associated with a genetic alteration of VEN metabolism.

Severity was driven by extreme VEN plasma concentrations. Schulz et al. define toxic levels as 800–1,500 ng/mL and coma/fatal ranges as 3,200–24,000 ng/mL (7). Six days after ingestion of an estimated dose of 9 g VEN, our patient’s plasma levels remained >20,000 ng/mL, within the lethal range and necessitating prolonged ICU care.

Clinical severity was reinforced by several well-recognized VEN-related toxicities, including serotonin syndrome, hypoglycaemia, and cardiotoxicity. A mild hepatic dysfunction and acute kidney injury (peak INR: 1.74 (Normal Values: 0,8-1,2); peak creatinine 6,55 mg/dL (NV: 0,6-1,3 mg/dL)) likely reduced drug clearance, thereby contributing to increased toxicity.

Serotonin syndrome is the most frequently reported severe VEN effect and occurs in overdose (8), and our patient met Hunter criteria (9) such as generalized hypertonic state with clonic movements on admission. The altered state of consciousness observed in our patient could also be aggravated by the bromazepam intake.

Hypoglycaemia has also been reported in overdoses (6). Our patient experienced hypoglycaemia for 72 hours, beginning 48 hours after admission, highlighting the severity of the overdose.

Cardiotoxicity, which may progress to cardiogenic shock, required prolonged inotropic support. Extracorporeal life support (ECMO) was not used, unlike in other reports (10).

To our knowledge, no previous case has described the simultaneous occurrence of all three complications during a single ICU stay after VEN overdose. These events resulted in a 21-day ICU stay and a 46-day total hospitalization, slightly longer than the 43-day course reported by Murphy in a VEN overdose case requiring ECMO (10).

**Figure 2:**
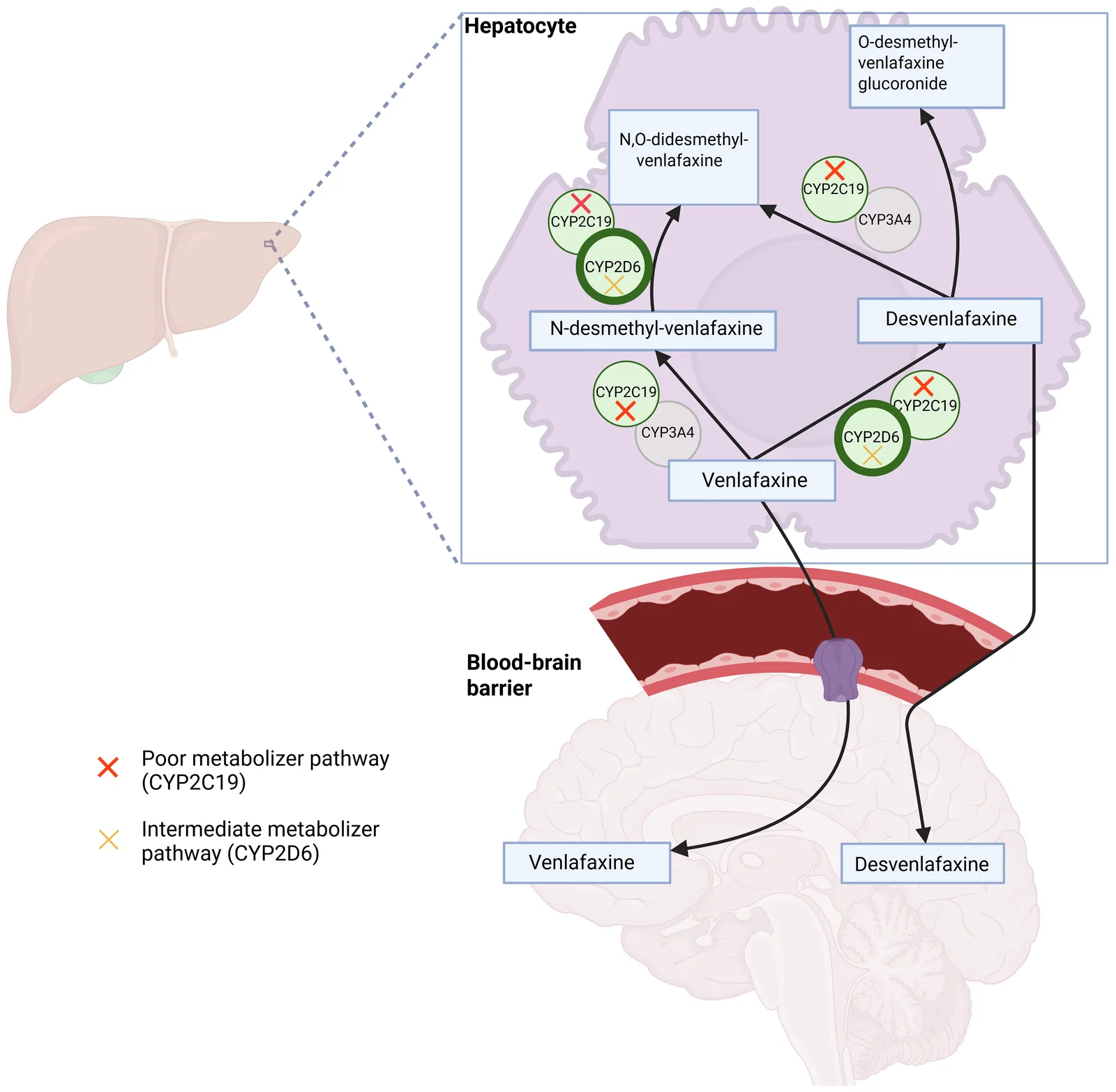
Schematic representation of venlafaxine metabolic pathways mediated primarily by CYP2D6, with additional contributions from CYP2C19 and CYP3A4, along with the resulting metabolites. Crosses indicate altered metabolic activity (poor [in red] or intermediate [in yellow] metabolizer status). Created using BioRender.com.

In light of the persistently elevated VEN and ODV levels on day 6, impaired metabolism due to pharmacogenetic variants appears to be the principal contributor to the unusually severe and prolonged toxicity. No drug interactions were found with her chronic medication or her hospital medication. This effect was likely exacerbated by concomitant hepatic and renal dysfunction, further reducing drug clearance and promoting accumulation. VEN undergoes hepatic metabolism to ODV primarily via CYP2D6 and CYP2C19, and to NDV via CYP2C19 and CYP3A4 (**Figure 2**).

Genetic polymorphisms affecting these enzymes may delay VEN elimination, prolonging critical illness in overdose cases. The O-desmethylvenlafaxine/venlafaxine ratio (ODV/VEN) can serve as a useful indicator of metabolic capacity. In individuals with normal CYP2D6 and CYP2C19 functions, the ODV/VEN ratio typically ranges from 2.7 to 7.7. In our patient, the low ODV/VEN ratio (0.17) indicates reduced formation of ODV. This could reflect impaired metabolism, potentially consistent with a poor metabolizer phenotype, although the contribution of multiple pathways warrant caution and limit definitive conclusions. Additionally this ratio may also be influenced by absorption kinetics, sampling time, dose-dependent nonlinear pharmacokinetic processes, and dynamic changes in organ function during critical illness, which may limit its interpretation in the setting of acute overdose. NDV was not measured because only VEN+ODV is pertinent for therapeutic drug monitoring due to non- clinical relevant activity of NDV (11).

Polymorphisms in metabolic pathways have been previously linked to severe toxicity. Vinetti et al. reported a severe acute cardiomyopathy following VEN overdose, attributed to the patient’s *intermediate metabolizer* phenotype for CYP2D6 (*1/*5) and to the total ingested VEN dose (i.e. 11.25 g). However, the patient exhibited a *rapid metabolizer* phenotype for CYP2C19 (*1/*17), which may have mitigated the toxicity of the overdose (12).

Jornil et al. reported the only known case of a patient with a combined *poor metabolizer* phenotype for both CYP2D6 and CYP2C19, unfortunately leading to a fatal outcome (13).

Our case appears to be the first illustrating the probable impact of a combined CYP2D6 *intermediate metabolizer* and CYP2C19 *poor metabolizer* phenotype on the complexity and duration of severe VEN overdose. This combination is rare, as the CYP2C19 *poor metabolizer* phenotype affects only 2–5% of the Caucasian population (14). and the CYP2D6 *intermediate metabolizer* phenotype affects 10-44% of global population (with only 3-5% of *5 allele in European population) (15). The expected frequency of this genotype combination corresponds to the product of the two individual frequencies, since they are located on independent genetic loci.

Pharmacogenetic testing is generally not expected to alter routine clinical management, and routine CYP genotyping is not recommended for all patients receiving VEN. However, in cases like the one reported here, identifying patients as poor or intermediate CYP2D6 metabolizers can be clinically relevant. Some expert groups, such as the Dutch Pharmacogenetics Working Group (DPWG), advise caution or recommend avoiding VEN in these patients due to a higher risk of adverse effects.

While the Clinical Pharmacogenetics Implementation Consortium (CPIC) recommendations focus exclusively on CYP2D6 genotype, recent data show that other variants, such as CYP2C19, can also meaningfully affect antidepressant metabolism and, in our opinion, should be considered. In this case, the patient’s impaired CYP2C19 pathway left the partially functional CYP2D6 pathway largely responsible for VEN biotransformation, contributing to drug accumulation alongside other factors discussed above.

An additional risk factor for increased VEN toxicity in patients with CYP2D6 genetic alterations is age, as older individuals tend to have higher VEN plasma concentrations (16). While previously reported cases involved patients under 45 years old, our patient was 73, which may have worsened the clinical presentation.

Given the large quantity of VEN XR tablets ingested, pharmacobezoar formation was considered a plausible contributing factor to the prolonged and markedly elevated VEN concentrations observed.

This phenomenon has been described in cases by Tuan Tiong and Kubovy (32.1 g of VEN XR for case 1, and 6.75 g of VEN XR for case 2) (17).

The detection of VEN in the gastric fluid on days 3 and 9, particularly in a patient without gastroparesis, led us to consider the presence of a pharmacobezoar as the most plausible working hypothesis. This interpretation was further supported by the patient’s secondary clinical deterioration, which may reflect delayed VEN release from the pharmacobezoar. In addition, serial serum VEN concentrations could have provided further support for this hypothesis by demonstrating ongoing or delayed systemic absorption. However, unlike the previously reported cases, we did not perform endoscopic evaluation because the detection of VEN in the day 9 gastric fluid was, at the time, considered sufficiently suggestive of a pharmacobezoar, and we therefore did not pursue additional investigations to document its presence. Consequently, neither the presence of a pharmacobezoar nor its contribution to the delayed clinical course can be definitively confirmed, representing a limitation of our report.

The lack of plasma VEN and metabolite measurements on admission and days before day 6 also restricts kinetic interpretation to the post-acute phase rather than the initial toxic window. It also prevents accurate characterization of absorption, peak exposure, and early elimination kinetics.

Although the elimination half-lives of VEN and ODV are expected to be prolonged in the presence of this combined genetic polymorphism, we did not calculate these half-lives. On the one hand, due to a lack of plasma samples on the day of patient admission and in the days that followed. On the other hand, because the likely presence of a pharmacobezoar is inconsistent with the notion of physiological prolonged half-lives.

The clinical utility of pharmacogenetic testing in the acute management of VEN overdose remains limited, as results are not available within the timeframe required to guide initial supportive care. The main value of pharmacogenetic assessment is diagnostic, providing an explanation for unusually severe, prolonged, or unexpected toxicity and identifying patients with altered VEN metabolism.

However, the implications of such findings extend beyond VEN, as CYP2D6 and CYP2C19 are involved in the metabolism of numerous other medications. Identification of an impaired metabolizer phenotype may therefore be relevant for future prescribing decisions, including dose optimization or selection of alternative therapies with lower risk of adverse effects. In selected cases of severe overdose with unexpectedly persistent drug concentrations or prolonged clinical evolution, a multidisciplinary implication (physician, pharmacist-biologist, clinical pharmacist) on pharmacogenetic testing may thus contribute to understanding individual susceptibility and informing subsequent therapeutic strategies.

## Conclusion

We present a case of complex and prolonged VEN overdose and its management in a patient with a combined phenotype of CYP2D6 *intermediate metabolizer* and CYP2C19 *poor metabolizer*.

Early measurement of VEN plasma concentrations from day 0 of the overdose, the ODV/VEN ratio and an understanding of VEN’s metabolic pathways are important tools for clinicians to identify kinetic abnormalities.

In addition to genotyping, performing digestive endoscopy and VEN concentration measurement on gastric fluid aspiration the days following the overdose may be considered in selected cases, particularly in cases of XR formulations, to investigate the presence of a pharmacobezoar.

## Data Availability

The original contributions presented in the study are included in the article/supplementary material, further inquiries can be directed to the corresponding author/s.

## References

[1] Sangkuhl K Stingl JC Turpeinen M Altman RB Klein TE . PharmGKB summary: venlafaxine pathway. Pharmacogenet Genomics. (2014) 24:62–72. doi: 10.1097/FPC.0000000000000003 24128936 PMC4098656

[2] Kringen MK Bråten LS Haslemo T Molden E . The influence of combined CYP2D6 and CYP2C19 genotypes on venlafaxine and O-desmethylvenlafaxine concentrations in a large patient cohort. J Clin Psychopharmacol. (2020) 40:137–44. doi: 10.1097/JCP.0000000000001174 32134850

[3] Thomas L De La Cruz CG Mata-Martín C González-Rodríguez I González-Martínez I Peñas-Lledó EM . Influence of CYP2D6, CYP2C19, and CYP2C9 pharmacogenetics and clinical factors on dose-normalized venlafaxine/O-desmethylvenlafaxine metabolic ratio in Spanish patients. Pharmaceuticals. (2026) 19:209. doi: 10.3390/ph19020209 41754751 PMC12944365

[4] Shams MEE Arneth B Hiemke C Dragicevic A Muller MJ Kaiser R . CYP2D6 polymorphism and clinical effect of the antidepressant venlafaxine. J Clin Pharm Ther. (2006) 31:493–502. doi: 10.1111/j.1365-2710.2006.00763.x 16958828

[5] Riley DS Barber MS Kienle GS Aronson JK von Schoen-Angerer T Tugwell P . CARE guidelines for case reports: explanation and elaboration document. J Clin Epidemiol. (2017) 89:218–35. doi: 10.1016/j.jclinepi.2017.04.026 28529185

[6] Francino M-C Deguigne MB Badin J Turcant A Perrotin D . Hypoglycaemia: a little known effect of venlafaxine overdose. Clin Toxicol. (2012) 50:215–7. doi: 10.3109/15563650.2012.660696 22372790

[7] Schulz M Schmoldt A Andresen-Streichert H Iwersen-Bergmann S . Revisited: therapeutic and toxic blood concentrations of more than 1100 drugs and other xenobiotics. Crit Care. (2020) 24:195. doi: 10.1186/s13054-020-02915-5 32375836 PMC7201985

[8] Kolecki P . Isolated venlafaxine-induced serotonin syndrome. J Emergency Med. (1997) 15:491–3. doi: 10.1016/S0736-4679(97)00078-4 9279702

[9] Dunkley EJC Isbister GK Sibbritt D Dawson AH Whyte IM . The Hunter Serotonin Toxicity Criteria: simple and accurate diagnostic decision rules for serotonin toxicity. QJM. (2003) 96:635–42. doi: 10.1093/qjmed/hcg109 12925718

[10] Murphy L Rasmussen J Murphy NG . Venlafaxine overdose treated with extracorporeal life support. CMAJ. (2021) 193:E167–70. doi: 10.1503/cmaj.201318 33526543 PMC7954576

[11] Hiemke C Bergemann N Clement H Conca A Deckert J Domschke K . Consensus guidelines for therapeutic drug monitoring in neuropsychopharmacology: update 2017. Pharmacopsychiatry. (2018) 51:9–62. doi: 10.1055/s-0043-116492 28910830

[12] Vinetti M Haufroid V Capron A Classen J-F Marchandise S Hantson P . Severe acute cardiomyopathy associated with venlafaxine overdose and possible role of CYP2D6 and CYP2C19 polymorphisms. Clin Toxicol. (2011) 49:865–9. doi: 10.3109/15563650.2011.626421 22077251

[13] Jornil J Nielsen TS Rosendal I Ahlner J Zackrisson AL Boel LWT . A poor metabolizer of both CYP2C19 and CYP2D6 identified by mechanistic pharmacokinetic simulation in a fatal drug poisoning case involving venlafaxine. Forensic Sci Int. (2013) 226:e26–31. doi: 10.1016/j.forsciint.2012.12.020 23332809

[14] Scott SA Sangkuhl K Gardner EE Stein CM Hulot J-S Johnson JA . Clinical Pharmacogenetics Implementation Consortium guidelines for cytochrome P450-2C19 (CYP2C19) genotype and clopidogrel therapy. Clin Pharmacol Ther. (2011) 90:328–32. doi: 10.1038/clpt.2011.132 21716271 PMC3234301

[15] Kane M . CYP2D6 overview: allele and phenotype frequencies Medical Genetics Summaries (2025). Bethesda (MD: National Center for Biotechnology Information (US. Available online at: https://www.ncbi.nlm.nih.gov/books/NBK574601/ (Accessed January 30, 2025).

[16] Waade RB Hermann M Moe HL Molden E . Impact of age on serum concentrations of venlafaxine and escitalopram in different CYP2D6 and CYP2C19 genotype subgroups. Eur J Clin Pharmacol. (2014) 70:933–40. doi: 10.1007/s00228-014-1696-8 24858822

[17] Ho TT Kubovy J . Endoscopic decontamination of massive, modified release venlafaxine overdose J Gastroenterol Res Pract (2024). Available online at: https://jjgastro.com/articles/JJGR-v4-1189.html (Accessed January 30, 2025).

